# Endothelin A receptor blocker and calcimimetic in the adenine rat model of chronic renal insufficiency

**DOI:** 10.1186/s12882-017-0742-z

**Published:** 2017-10-27

**Authors:** Suvi Törmänen, Ilkka Pörsti, Päivi Lakkisto, Ilkka Tikkanen, Onni Niemelä, Timo Paavonen, Jukka Mustonen, Arttu Eräranta

**Affiliations:** 1 Faculty of Medicine and Life Sciences, University of Tampere, Tampere, Finland; 2 Department of Internal Medicine, Tampere University Hospital, Tampere, Finland; 3 Minerva Institute for Medical Research, Helsinki, Finland; 4 Clinical Chemistry and Hematology, University of Helsinki and Helsinki University Hospital, Helsinki, Finland; 5 Abdominal Center, Nephrology, University of Helsinki and Helsinki University Hospital, Helsinki, Finland; 6 Medical Research Unit, Seinäjoki Central Hospital, Seinäjoki, Finland; 7 Fimlab Laboratories, Tampere, Finland; 8 School of Medicine / Internal Medicine, FIN-33014 University of Tampere, Tampere, Finland

**Keywords:** Chronic kidney disease, Sitaxentan, Cinacalcet, Creatinine, Parathyroid hormone, Renal renin-angiotensin system

## Abstract

**Background:**

We studied whether endothelin receptor antagonist and calcimimetic treatments influence renal damage and kidney renin-angiotensin (RA) components in adenine-induced chronic renal insufficiency (CRI).

**Methods:**

Male Wistar rats (*n* = 80) were divided into 5 groups for 12 weeks: control (*n* = 12), 0.3% adenine (Ade; *n* = 20), Ade + 50 mg/kg/day sitaxentan (*n* = 16), Ade + 20 mg/kg/day cinacalcet (n = 16), and Ade + sitaxentan + cinacalcet (n = 16). Blood pressure (BP) was measured using tail-cuff, kidney histology was examined, and RA components measured using RT-qPCR.

**Results:**

Adenine caused tubulointerstitial damage with severe CRI, anemia, hyperphosphatemia, 1.8-fold increase in urinary calcium excretion, and 3.5-fold and 18-fold increases in plasma creatinine and PTH, respectively. Sitaxentan alleviated tubular atrophy, while sitaxentan + cinacalcet combination reduced interstitial inflammation, tubular dilatation and atrophy in adenine-rats. Adenine diet did not influence kidney angiotensin converting enzyme (ACE) and AT_4_ receptor mRNA, but reduced mRNA of renin, AT_1a_, AT_2_, (pro)renin receptor and Mas to 40–60%, and suppressed ACE2 to 6% of that in controls. Sitaxentan reduced BP by 8 mmHg, creatinine, urea, and phosphate concentrations by 16–24%, and PTH by 42%. Cinacalcet did not influence BP or creatinine, but reduced PTH by 84%, and increased hemoglobin by 28% in adenine-rats. The treatments further reduced renin mRNA by 40%, while combined treatment normalized plasma PTH, urinary calcium, and increased ACE2 mRNA 2.5-fold versus the Ade group (*p* < 0.001).

**Conclusions:**

In adenine-induced interstitial nephritis, sitaxentan improved renal function and tubular atrophy. Sitaxentan and cinacalcet reduced kidney renin mRNA by 40%, while their combination alleviated tubulointerstitial damage and urinary calcium loss, and increased kidney tissue ACE2 mRNA.

**Electronic supplementary material:**

The online version of this article (10.1186/s12882-017-0742-z) contains supplementary material, which is available to authorized users.

## Background

Chronic kidney disease (CKD) and its progression to end stage renal disease (ESRD) remain a global clinical challenge [1, 2]. Regardless of the original kidney insult, one of the major causes leading to the decline of renal function is interstitial fibrosis [3, 4]. Adenine diet administration to rats, and the subsequent deposition of 2,8-dihydroxyadenine crystals in kidney tissue, induce an inflammatory response resembling the pathology of interstitial nephritis and the consequent fibrosis [5–7]. The adenine model has been shown to result in severe chronic renal insufficiency (CRI) with its typical uremic findings such as elevated creatinine and urea, anemia, and secondary hyperparathyroidism (SHPT) [5, 8–11].

Activation of the renin-angiotensin system (RAS) is, by far, the best-characterized promoter of inflammation and fibrosis in the pathology of CRI [12, 13]. The formation of local angiotensin II is known to promote inflammation and fibrosis [12, 13], while inhibition of RAS attenuates proteinuria, glomerulosclerosis and also the development of interstitial fibrosis [14]. However, the need for additional renoprotective therapeutics is evident, and thus the endothelin system has recently garnered high interest.

The endothelin system is activated in virtually all causes of CKD, in which endothelin receptor A (ETA) activation promotes vasoconstriction, renal cell injury, inflammation, and fibrosis. The endothelin system is also linked to RAS by positive feedback loops [15–17]. While ETA antagonists have been shown to ameliorate renal injury, fibrosis, proteinuria, and disease progression in experimental diabetic, hypertensive, and remnant kidney rat models of CKD [16], less is known about the effects of ETA antagonism in renal diseases of tubulointerstitial origin. Some studies suggest that ETA antagonism might deteriorate renal function in polycystic models of CKD [18].

Disturbed calcium-phosphate metabolism and SHPT may also contribute to the progression of CKD [19]. We previously found that dietary phosphate loading, and phosphate binding by oral calcium carbonate treatment, altered the contents of RAS components in the kidney and aorta, and also influenced glomerulosclerosis and tubulointerstitial damage in 5/6 nephrectomized rats [20–22]. However, the key player modulating RAS components through changes in calcium-phosphate metabolism has remained elusive. Recently, reducing serum parathyroid hormone (PTH) with cinacalcet, a positive allosteric modulator of the calcium sensing receptor (CaSR), in adenine-induced rat model of CKD was reported to attenuate renal fibrogenesis and reduce plasma creatinine concentration [10]. It is plausible that some of the effects of oral calcium carbonate supplementation and the positive allosteric modulation of CaSR, i.e. calcimimetics, might be mediated via local CaSR activation.

Recently, major interest has been directed towards the possible benefits of endothelin type A receptor blockade in kidney diseases with glomerular damage and proteinuria [16]. Here we evaluated the effects of treatment with the selective ETA antagonist sitaxentan and the positive allosteric CaSR agonist cinacalcet, alone and in combination, on the progression of adenine rat model of severe interstitial nephritis. As both endothelin system and calcium-phosphate metabolism can affect intrarenal RAS, we hypothesized that the putative positive outcomes of sitaxentan and cinacalcet might be reflected as changes in the kidney components of RAS, transforming growth factor-ß1 (TGF-ß1) and connective tissue growth factor (CTGF), key markers and potential therapeutic targets of kidney fibrosis [23]. To our knowledge, the local kidney tissue RAS components have not been previously studied in this model of CKD.

## Methods

### Animals and experimental design

Eighty male Wistar rats (Harlan Laboratories, Horst, The Netherlands) aged 8 weeks were housed four to a cage with free access to water and chow. At the age of 10 weeks the rats were divided into 5 groups with matched systolic blood pressures (BP) and body weights. The systolic BP was measured at +28°C by the tail-cuff method (Model 129 Blood Pressure Meter; IITC Inc., Woodland Hills, CA).

One group was given the control diet (RM3, Scanbur, Karlslunde, Denmark) and 4 groups received 0.3% adenine (Sigma-Aldrich, Saint Louis, MO) added to the chow. We chose the continuous 0.3% adenine diet for this 12-week study [11], based on (1) previous findings of high mortality related to the 0.75% adenine diet, even if adenine was withdrawn after 4 weeks of administration [24]; and (2) concerns about the reversibility of CRI when adenine administration is discontinued [9, 24].

In addition to adenine, rats in 3 groups received either sitaxentan (50 mg/kg/d added to the drinking water protected from light; Pfizer, New York, NY), cinacalcet (20 mg/kg/d added to the 0.3% adenine chow; Amgen Thousand Oaks, CA), or both of these treatments for 12 weeks. The doses of these compounds were chosen on the basis of previous reports [10, 25–29]. The study groups were: control (Control, *n* = 12), adenine (Ade, *n* = 20), adenine + sitaxentan (Ade + S, *n* = 16), adenine + cinacalcet (Ade + C, n = 16), and adenine + combination treatment (Ade + SC, n = 16). The medications were continued until the end of the study.

Body weights were monitored weekly, systolic BP was measured at the end of the study, and 24-h water consumption, urine output, and chow consumption were monitored twice during the treatments (weeks 5 and 10) in metabolic cages. Urine samples were stored at −70°C for analyses. At close of the study, rats were anesthetized with intraperitoneal urethane (1.3 g/kg), the carotid artery was cannulated, and blood samples were drawn with EDTA and heparin as anticoagulants, as appropriate. Plasma and weighed tissue samples were stored at −70 °C.

The experimental design was approved by the Animal Experimentation Committee of the University of Tampere, and the Provincial Government of Western Finland, Department of Social Affairs and Health, Finland; identification number ESLH-2008-03943/Ym-23. The investigation conforms to the Guiding Principles for Research Involving Animals.

### Sample size calculation

As the major aim of the study was to evaluate if the treatments can ameliorate adenine-induced CRI, creatinine was chosen as the primary outcome variable. The relevant difference in the means of creatinine values of treated and untreated adenine rats as well as the standard deviation of creatinine values were both estimated at 40 μmol/l. The estimation of standard deviation was derived from previously published adenine rat experiments [5]. Power of the study was set at 0.80 and the type I error probability at 0.05. The sample size of untreated adenine rats was adjusted for expected attrition of 20% and, thus, the sample size was multiplied by 1.25 in the Ade group. The acquired sample sizes were 15 and 19 for treated and untreated adenine rats, respectively, and were ultimately rounded up to the nearest even number (rats were housed four to a cage). The sample size was calculated using PS program [30].

### Hormonal and chemical analyses

Sodium, potassium, creatinine, urea, phosphate, calcium, cholesterols and proteins were measured using standard clinical chemical methods (Cobas Integra 800 Clinical Chemical Analyzer, Roche Diagnostics, Basel, Switzerland). The determination of 24-h creatinine clearance was based on urine determinations from week 10 and plasma samples taken at close of the study. The enzymatic method used here for creatinine determination has been previously shown to be reliable in both healthy and diseased rats [31]. Hemoglobin was determined photometrically (Technicon H*2™, Technicon Instruments Corporation, Tarrytown, NY) and rat intact parathyroid hormone (PTH) levels by immunoradiometric assay (Immutopics Inc. San Clemente, CA).

### Renal histology

Morphological investigations from haematoxylin-eosin stained kidney sections were performed with light microscopy in a blinded manner. Glomerular and tubulointerstital scores were determined according to Schwarz et al. [32], originally described by El Nahas et al. [33] and Veniant et al. [34], respectively.

Semi-quantitative glomerular score for each group was assessed by examining 100 glomeruli at magnifications ×100 and ×400: grade 0, normal glomeruli; grade 1, mesangial expansion; grade 2, mild/moderate segmental hyalinosis/sclerosis involving <50% of the glomerular tuft; grade 3, diffuse glomerular hyalinosis/sclerosis involving ≥50% of the tuft; grade 4, diffuse glomerulosclerosis with total tuft obliteration and collapse. The index in each group was expressed as the mean of all scores obtained.

The tubulointerstitial scores (tubular atrophy, tubular dilatation, interstitial expansion, interstitial inflammation and interstitial fibrosis) were determined by visualizing randomly selected ten fields per kidney at a magnification ×100. The grading was as follows: grade 0, no changes; grade 1, lesions involving <25% of the area; grade 2, lesions involving 25–50%; grade 3, lesions affecting >50% of the area; and grade 4, lesions involving (almost) the entire area. The means of all scores given comprised the tubulointerstitial indexes.

### Real-time quantitative RT-PCR

Total RNA was isolated from kidney tissue using Trizol reagent (Invitrogen, Carlsbad, CA). Reverse transcription of RNA was performed using M-MLV reverse transcriptase (Invitrogen) according to the manufacturer’s instructions. Beta-actin was used as a housekeeping gene. PCRs were performed with SYBR Green or TaqMan chemistry using ABI PRISM 7000 sequence detection (Applied Biosystems, Foster City, CA).

PCRs for angiotensin converting enzyme (ACE), angiotensin II receptor type 1_a_ (AT_1aR_), angiotensin IV receptor (AT_4R_), (pro)renin receptor (PRR), and TGF-ß1 were performed in duplicate in 25 μl final volume containing 1X SYBR Green Master mix (Applied Biosystems) and 300 nM of primers. PCRs for ACE2, angiotensin II receptor type 2 (AT_2R_), renin, and CTGF were performed in duplicate in 25 μl final volume containing 1X TaqMan Master mix (Applied Biosystems), 300 nM of primers (for renin 900 nM) and 100 nM of ACE2, 150 nM of AT_2R_, 250 nM of renin, or 200 nM of CTGF TaqMan probe, respectively.

PCR cycling conditions for mRNAs were 10 min at +95 °C and 40 cycles of 20 s at +95 °C and 1 min at +60 °C. Data were analyzed using the absolute standard curve method [35]. Beta-actin (TaqMan assay code Rn00667869_m1) was analyzed in a similar fashion to 18S analysis in our earlier paper [20]. The expressions of beta-actin did not differ between the groups, and the other results were normalized for the levels of beta-actin.

### Western blotting of TGF-ß1

Kidney tissues were homogenized in a lysis buffer containing 100 mmol/L NaCl, 10 mmol/L KCl, 8 mmol/L Na_2_HPO_4_, 3 mmol/L MgCl_2_, 0.5% NP40, 10 mmol/L Tris-HCl, pH 7.4 and protease inhibitors (CompleteTM Mini, Protease Inhibitor Cocktail Tablets, Roche Diagnostics GmbH, Mannheim, Germany). Homogenates were incubated on ice for 30 min and centrifuged at 15000 g for 15 min. Protein concentration in the supernatant was determined using the BCA protein assay kit (Pierce, Rockford, IL, USA). Equal amounts of protein (25 μg) were fractionated by 4–20% Mini-PROTEAN TGX Stain-Free Gel (Bio-Rad, Hercules, CA, USA). The gel was stain-free activated and blotted onto a low-fluorescence polyvinylidene fluoride membrane (LF PVDF Trans-Blot Turbo RTA Transfer Kit) with Trans-Blot Turbo Transfer System (Bio-Rad). The membrane was then stain-free imaged for total protein normalization using ChemiDoc Touch Imaging System (Bio-Rad) and probed with mouse anti-TGF-ß1 (R&D Systems, Minneapolis, MN, USA). Enhanced chemiluminescent detection was performed using the SuperSignal West Pico Chemiluminescent Substrate (Thermo Scientific, Rockford, IL, USA) and the chemiluminescent application on the ChemiDoc Touch Imaging System. Quantification of signal intensities was performed using the ImageLab software (Bio-Rad) by normalizing the intensities of specific bands to the total protein content on the membrane. Representative original Western blot image of TGF-ß1 is shown in Additional file 1: Figure S1.

### Data presentation and analysis of results

Statistical analysis was carried out using one-way analysis of variance (ANOVA), and post-hoc comparisons were performed with the Tukey HSD test if the variables had equal variances verified by the Levene’s test. If this criterion was not met, the Kruskal-Wallis test and the Mann-Whitney test with the Bonferroni correction were used. The results in the table were presented as mean ± SEM, while the figures show mean ± 95% confidence interval of the mean. Differences were considered significant when *P* < 0.05. SPSS 17.0 software (SPSS Inc., Chicago, IL, USA) was used for the statistics.

## Results

### Animal data

The adenine diet resulted in decreased body weights and lower chow intake, but no rats were lost during the study (Table 1). Chow intake in the Ade + S group was reduced slightly less than in the Ade group. The adenine diet did not elevate systolic BP, whereas both sitaxentan-treated groups showed reduced BP when compared with the Ade group. The volumes of drinking fluid and urine were increased in all adenine groups, while the Ade + SC group showed lowest urine volume among the adenine-treated groups.

**Table 1:** Animal data and laboratory findings in the study groups

	Control	Ade	Ade + S	Ade + C	Ade + SC
Number of animals	12	20	16	16	16
Body weight (g)					
week 0	406 ± 8	409 ± 5	409 ± 5	409 ± 5	409 ± 5
week 12	450 ± 11	339 ± 6*	341 ± 6*	340 ± 7*	359 ± 8*
Chow intake at week 10 (g/24 h)	20.3 ± 1.0	13.9 ± 0.3*	16.5 ± 0.5*†	15.1 ± 0.7*	14.8 ± 0.8*
Systolic blood pressure (mmHg)					
week 0	139 ± 1	138 ± 1	139 ± 1	138 ± 1	139 ± 1
week 9	137 ± 2	138 ± 1	130 ± 2†	138 ± 1‡	129 ± 2*†
Drinking volume (ml/24 h)	31.0 ± 1.9	87.5 ± 2.9*	88.1 ± 1.8*	83.5 ± 3.5*	81.2 ± 2.4*
Urine volume (ml/24 h)	20.0 ± 2.3	73.9 ± 2.2*	82.7 ± 2.7*‡	69.1 ± 3.8*‡	54.7 ± 2.5*†
24-h creatinine clearance (μl/min/kg)^a^	379 ± 23	82 ± 6*	119 ± 8*†	96 ± 11*	122 ± 13*†
Final plasma					
Sodium (mmol/l)	135.1 ± 0.7	138.3 ± 0.5*	141.5 ± 0.6*†	140.1 ± 0.5*	140.7 ± 0.5*†
Potassium (mmol/l)	3.63 ± 0.09	4.34 ± 0.07*	4.22 ± 0.08*	4.19 ± 0.12*	4.05 ± 0.07*
Cholesterol (mmol/l)	1.45 ± 0.05	2.32 ± 0.10*	1.78 ± 0.09†	1.99 ± 0.10*‡	1.61 ± 0.05†
HDL (mmol/l)	0.60 ± 0.02	0.91 ± 0.04*	0.70 ± 0.02†	0.82 ± 0.04*	0.69 ± 0.02*†
Trigly (mmol/l)	1.06 ± 0.14	1.14 ± 0.10	0.86 ± 0.10	0.98 ± 0.08‡	0.68 ± 0.04†
Non-HDL (mmol/l)	0.86 ± 0.04	1.41 ± 0.07*	1.08 ± 0.08†	1.17 ± 0.07*‡	0.92 ± 0.04†
Proteins (g/l)	53.4 ± 1.0	51.9 ± 0.8	52.8 ± 1.2	51.1 ± 1.5	51.9 ± 0.9

Mean ± SEM; Ade, 0.3% adenine diet; S, sitaxentan 50 mg/kg/day; C, cinacalcet 20 mg/kg/day; ^a^estimated from urine collection during week 10 and final plasma creatinine; *p < 0.05 vs. Control; †*p* < 0.05 vs. Ade; ‡*p* < 0.05 vs. Ade + SC

### Laboratory findings

**Fig. 1:**
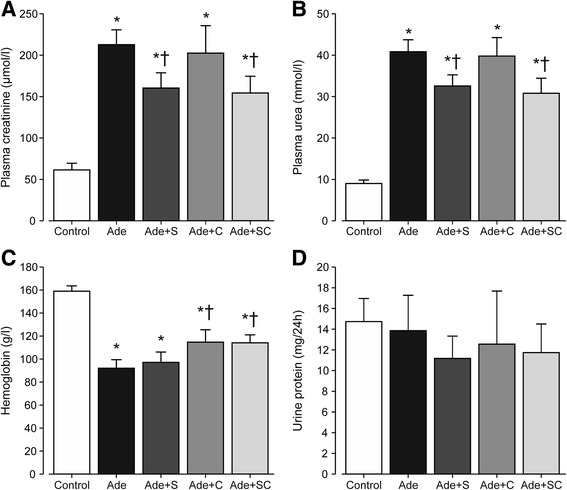
Bar graphs show plasma creatinine (**a**), urea (**b**), blood hemoglobin (**c**), and 24-h urine protein excretion (**d**) in rats ingesting normal diet (Control; *n* = 12), 0.3% adenine diet (Ade; *n* = 20), Ade + 50 mg/kg/day sitaxentan (Ade + S; *n* = 16), Ade + 20 mg/kg/day cinacalcet (Ade + C; n = 16), and Ade + sitaxentan + cinacalcet (Ade + SC; n = 16); mean ± confidence interval; ^*^ *P* < 0.05 vs. Control, ^†^ *P* < 0.05 vs. Ade, ‡*P* < 0.05 vs. Ade + SC

All adenine-diet groups showed markedly elevated plasma creatinine and urea concentrations (Fig. 1a-b). In the Ade group plasma creatinine and urea were 3.5 and 4.5-fold higher, respectively, than in the Control group. Sitaxentan treatment alone and in combination with cinacalcet ameliorated the increase in creatinine and urea when compared with the Ade group. Creatinine clearance in the Ade group was reduced to about 22%, and in the sitaxentan-treated groups to about 32%, of that in the Control group (Table 1). Cinacalcet treatment alone did not influence plasma creatinine or urea concentrations versus the Ade group (Fig. 1a and b).

Hemoglobin was markedly reduced in the Ade group when compared with the Control group (Fig. 1c). Sitaxentan treatment did not affect hemoglobin, while cinacalcet treatment alone and in combination with sitaxentan elevated hemoglobin by 28% when compared with the Ade group. There were no differences in 24-h urine protein excretion among the study groups (Fig. 1d).

Plasma sodium and potassium concentrations were increased in all adenine groups, while plasma sodium level was slightly further elevated by sitaxentan (Table 1). Plasma total, HDL, and non-HDL cholesterol concentrations were increased in the Ade group, and were not significantly influenced by cinacalcet treatment. However, plasma total and non-HDL cholesterol concentrations did not differ from the Control group in the sitaxentan-treated groups. Plasma protein concentrations did not differ between any of the study groups (Table 1).

**Fig. 2:**
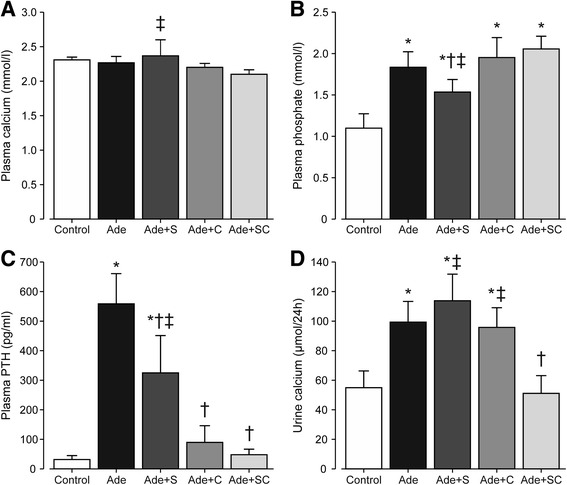
Bar graphs show plasma calcium (**a**), phosphate (**b**), PTH (**c**), and 24-h urine calcium excretion (**d**); study groups as in Fig. 1; mean ± confidence interval; ^*^ *P* < 0.05 vs. Control, ^†^ *P* < 0.05 vs. Ade, ‡*P* < 0.05 vs. Ade + SC

Plasma total calcium concentration was not affected by the adenine diet (Fig. 2a), but was slightly higher in the Ade + S than in the Ade + SC group. Plasma phosphate was significantly increased in all adenine-fed rats (Fig. 2b), while the Ade + S rats showed lower phosphate levels than the Ade + SC rats. Plasma PTH was increased 18-fold in the Ade group when compared with the Control group (Fig. 2c). Sitaxentan treatment alone reduced plasma PTH concentration by 42% when compared with the Ade group, while plasma PTH in the two cinacalcet-treated groups did not significantly differ from that in the Control group. The 24-h urinary calcium excretion was increased about 2-fold in all other adenine groups versus the Control group, with the exception of the combined sitaxentan + cinacalcet group, in which urinary calcium excretion did not differ from that in the Control group (Fig. 2d).

### Renal histology

**Fig. 3:**
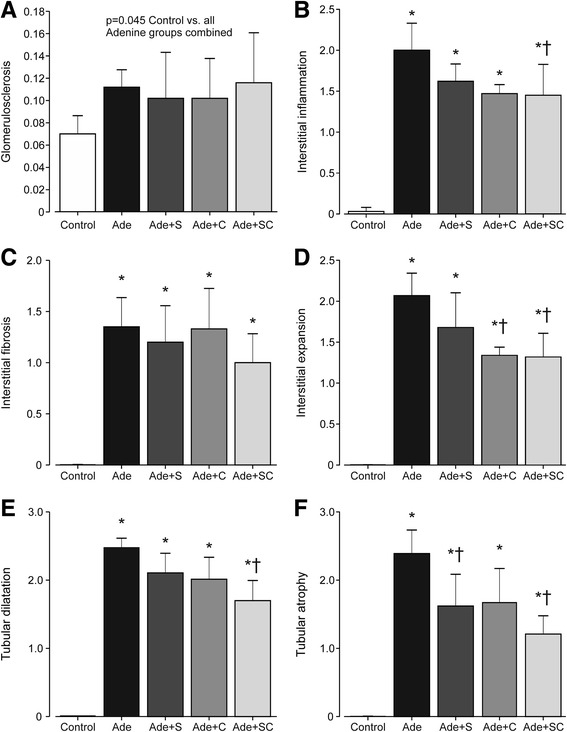
Bar graphs show indices of glomerulosclerosis (**a**), interstitial inflammation (**b**), interstitial fibrosis (**c**), interstitial expansion (**d**), tubular dilatation (**e**), and tubular atrophy (**f**); study groups as in Fig. 1; mean ± confidence interval; ^*^ *P* < 0.05 vs. Control, ^†^ *P* < 0.05 vs. Ade

**Fig. 4:**
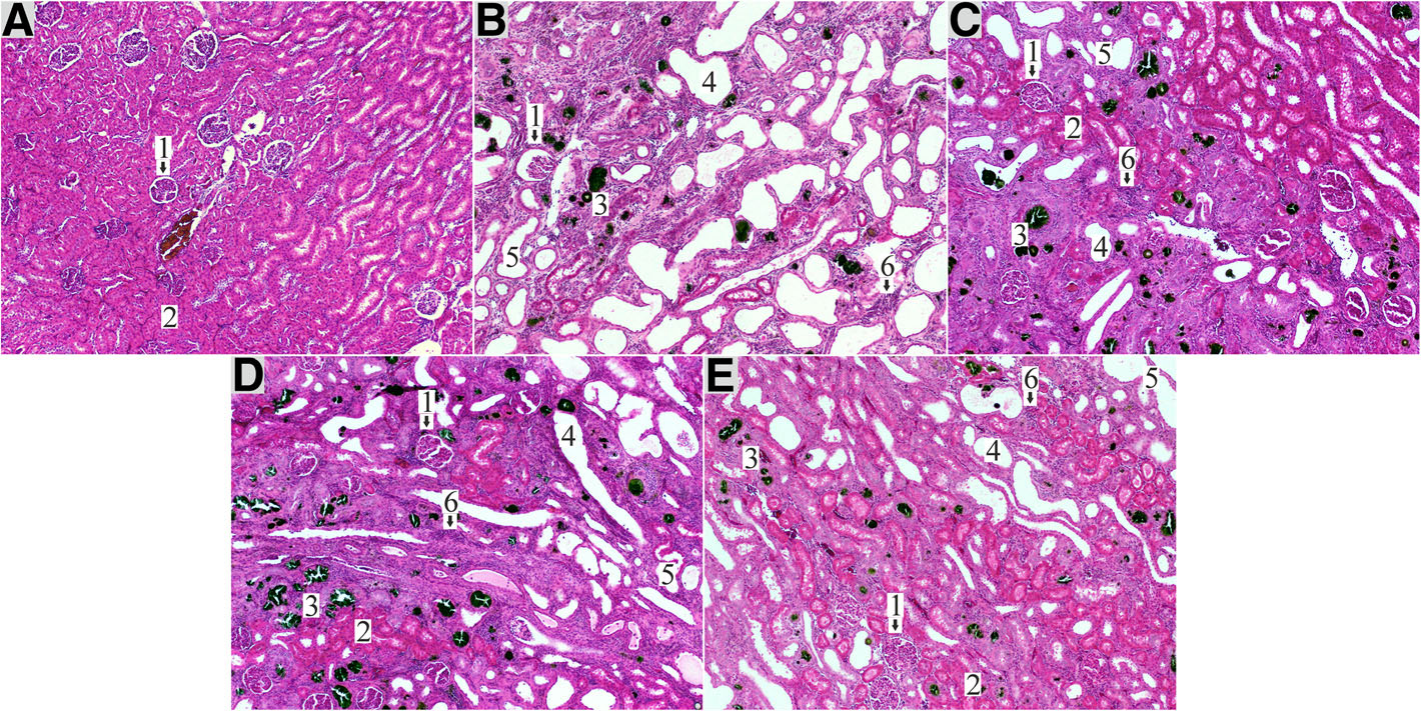
Representative photomicrographs of haematoxylin-eosin stained kidney histology in the study groups; Control (**a**), Ade (**b**), Ade + S (**c**), Ade + C (**d**), Ade + SC (**e**); 1 = glomeruli, 2 = healthy tissue (tubules); 3 = adenine crystal formations; 4 = dilated tubule; 5 = tubular atrophy; 6 = interstitial inflammation

The deposits of 2,8-dihydroxyadenine crystals were accompanied by clear tubulointerstitial damage in all adenine-fed rats (Figs. 3 and 4). In contrast, the glomeruli of the adenine groups showed only minor alterations, so that the glomerulosclerosis index was slightly higher in all adenine groups combined than in the Control group (Fig. 3a).

The adenine diet increased interstitial infiltration of inflammatory cells when compared with the Control group (Figs. 3b and 4). The combination of sitaxentan and cinacalcet alleviated interstitial inflammation when compared with untreated Ade rats, while the influence of cinacalcet monotherapy was not significant (*p* = 0.054 for Ade + C vs. Ade). Interstitial fibrosis was moderately increased in all adenine-fed rats, and was not affected by the treatments (Figs. 3c and 4). The adenine diet induced expansion of the interstitial tissue (reflecting inflammation, fibrosis and edema), while cinacalcet alone and in combination with sitaxentan alleviated interstitial tissue expansion (Figs. 3d and 4).

All adenine groups showed increased scores of tubular dilatation and atrophy when compared with the Control group, while the combination of cinacalcet and sitaxentan alleviated both tubular dilatation and atrophy. Sitaxentan treatment alone also alleviated tubular atrophy, while the effect of cinacalcet monotherapy was not significant (*p* = 0.095) (Figs. 3e-f and 4)

### Renal RAS, CTGF and TGF-ß1 mRNAs, and TGF-ß1 protein

**Fig. 5:**
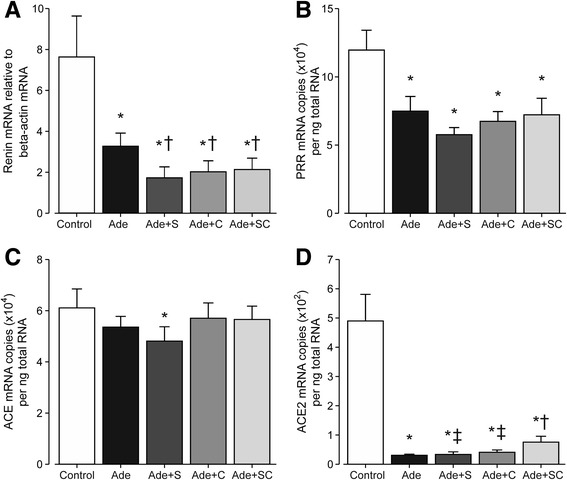
Bar graphs show renin (**a**), (pro)renin receptor (**b**), angiotensin converting enzyme (**c**), and angiotensin converting enzyme 2 (**d**) mRNA contents in kidney tissue determined using real-time RT-qPCR; renin mRNA expression (**a**) is shown relative to beta-actin expression; study groups as in Fig. 1; mean ± confidence interval; ^*^ *P* < 0.05 vs. Control, ^†^ *P* < 0.05 vs. Ade, ‡*P* < 0.05 vs. Ade + SC

Renal renin mRNA levels were strongly reduced by the adenine diet, while the treatments resulted in further 40–45% reduction in renin mRNA when compared with the Ade group (Fig. 5a). PRR mRNA contents were reduced in all adenine-fed groups by about 40–50%, but no additional effect was observed with the treatments (Fig. 5b). Renal ACE mRNA content was not affected by adenine administration, but was moderately reduced in the Ade + S group versus the Control group (Fig. 5c). In contrast, kidney ACE2 mRNA content was strikingly suppressed (by 85–94%) in all adenine groups when compared with the Control group (Fig. 5d). The suppression of ACE2 mRNA, however, was less marked in the group treated with sitaxentan + cinacalcet when compared with the other adenine groups, and ACE2 content in the Ade + SC group was 2.5-fold higher than in the Ade group (*p* < 0.001).

**Fig. 6:**
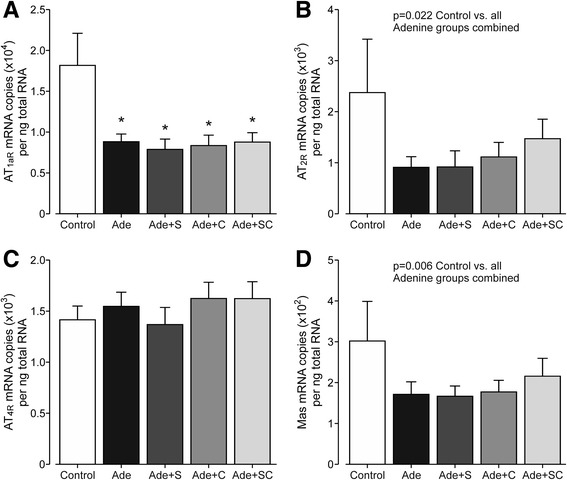
Bar graphs show angiotensin II receptor type 1_a_ (**a**), angiotensin II receptor type 2 (**b**), angiotensin II receptor type 4 (**c**), and Mas oncogene (**d**) mRNA contents in kidney tissue determined using real-time RT-qPCR; study groups as in Fig. 1; mean ± confidence interval; ^*^ *P* < 0.05 vs. Control, ^†^ *P* < 0.05 vs. Ade, ‡*P* < 0.05 vs. Ade + SC

Renal AT_1aR_ mRNA content was decreased by about 50% in all adenine-diet groups when compared with the Control group (Fig. 6a). Kidney AT_2R_ mRNA was also reduced by about 50% in the adenine groups (Fig. 6b), although significant difference was not reached between any single group versus the Control group. AT_4R_ mRNA remained unchanged by adenine feeding (Fig. 6c). Mas receptor mRNA in the adenine groups was about 40% lower than that in the Control group (Fig. 6d), while again no significant difference was observed between any single group versus the Control group. Neither sitaxentan nor cinacalcet treatment influenced renal tissue AT_1aR_, AT_2R_, AT_4R_, or Mas mRNA contents (Fig. 6a-d).

**Fig. 7:**
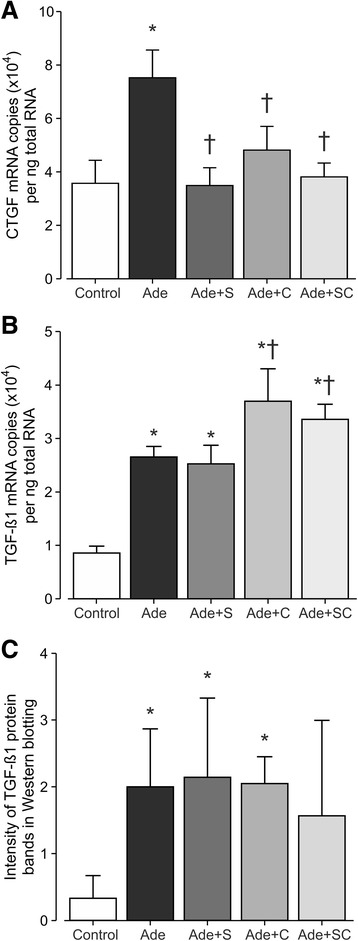
Bar graphs show connective tissue growth factor (**a**) and transforming growth factor-ß1 (**b**) mRNA contents determined in kidney tissue using real-time RT-qPCR; and transforming growth factor-ß1 protein content determined using Western blotting (**c**); study groups as in Fig. 1; mean ± confidence interval; ^*^ *P* < 0.05 vs. Control, ^†^ *P* < 0.05 vs. Ade

CTGF mRNA content was increased in the Ade group, while all treatments restored CTGF mRNA to the level of the Control rats (Fig. 7a). TGF-ß1 mRNA content was increased in the Ade and Ade + S groups, and slightly further elevated in the two cinacalcet-treated Ade-groups (Fig. 7b). When determined using Western blotting, TGF-ß1 protein was clearly increased by the adenine-diet, and was not affected by sitaxentan or cinacalcet treatments. The difference in kidney tissue TGF-ß1 protein content was not significant between the Control and Ade + SC groups due to the large variability in the TGF-ß1 signal in the latter group (Fig. 7c).

## Discussion

This study investigated the effects of sitaxentan and cinacalcet, alone and in combination, on the progression of CRI and kidney RAS components in the adenine rat model of CRI. The 0.3% adenine diet for 12 weeks induced severe CRI corresponding to stage 4 CKD, with typical uremic findings including anemia, hyperphosphatemia, SHPT, and elevated plasma potassium concentration [36]. The rats receiving adenine also showed weight loss, increased water consumption and polyuria, in concert with previous findings in this model [5, 10, 11]. However, systolic BP and urinary protein excretion were not increased. We applied the tail-cuff method to measure systolic BP, and with this approach we have previously found elevated BP in the 5/6 nephrectomy model of CRI [20–22]. By means of radiotelemetry, a more accurate method to measure BP, a moderate 18 mmHg elevation of mean arterial pressure was reported in adenine-fed rats [37]. However, the level of CRI in those rats corresponded to stage 5 CKD with 90% reduction in creatinine clearance [36], whereas in the present study the reduction was approximately 80%. Increased proteinuria has previously been reported in rats fed 0.75% adenine diet for 4 weeks, followed by the withdrawal of adenine for 8 weeks, however, in that study the rats also had severe stage 5 CKD with a 10-fold increase in plasma creatinine [10]. Thus, the above discrepancies can be partially explained by variations in the level of renal insufficiency due to the different protocols of adenine administration. Altogether, the absence of proteinuria and the present morphological findings showing tubulointerstitial tissue damage following adenine administration indicate that this model is associated with low level of glomerular damage. This notion is supported by similar histopathological findings in previous rat and mice adenine models of CKD [5, 38]. Of note, in the present study plasma protein concentrations were similar in all rat groups, and this argues against the view that the adenine-diet would have caused marked extracellular fluid accumulation.

The present study showed that selective ETA antagonism with sitaxentan at 50 mg/kg/day moderately improved renal function in this non-proteinuric model of advanced interstitial nephritis. The facts that a similar improvement in creatinine clearance was observed in both sitaxentan-treated groups, and that no rats were lost during the study, strengthen this finding. Sitaxentan also reduced tubular atrophy, while the combination of sitaxentan + cinacalcet reduced interstitial inflammation, interstitial expansion, tubular dilatation and tubular atrophy in the adenine-treated rats. The inhibition of the direct effects of the ETA receptor is the obvious mechanism for the reduced progression of CRI and improved morphology in the sitaxentan-treated rats, as ETA activation is known to promote renal cell injury, inflammation and fibrosis [16, 17]. Previously treatment with endothelin receptor antagonists has ameliorated renal injury, fibrosis, proteinuria, and disease progression in experimental diabetic, hypertensive, and remnant kidney rat models of CKD [16]. Our results indicate that selective ETA antagonism is also beneficial in this interstitial model of chronic nephritis.

Although the present model of CRI was not hypertensive, sitaxentan administration reduced BP. As BP was reduced to levels lower than in normal controls in spite of the prevailing CRI, this effect was rather attributed to the direct effects of ETA antagonism than to the beneficial influences on renal function. Due to the vasodilating effects, sitaxentan influences kidney hemodynamics [15–17], and the beneficial effects on renal function may have partially been mediated via increased renal blood flow. The possibility remains that sitaxentan interfered with the adenine-induced renal injury via its effects on kidney blood flow and tubular function, as such mechanisms may have subsequently influenced the formation of adenine crystals in the tubular fluid. Sitaxentan treatment also reduced plasma phosphate, PTH, total cholesterol, and non-HDL cholesterol concentrations. The disturbances of calcium-phosphate metabolism are associated with the level of impairment in kidney function [36]. In addition, CRI is characterized by abnormalities in the composition and metabolism of plasma lipoproteins [39, 40], and a significant reduction of lipoprotein catabolism is observed in advanced renal insufficiency [40]. Therefore, the beneficial effects of sitaxentan on phosphate, PTH and lipid metabolism were probably due to the beneficial influence on renal function.

As expected, cinacalcet treatment at 20 mg/kg/day effectively reduced PTH, while the treatment was without influence on renal function in the adenine model of CRI. Disturbed calcium-phosphate metabolism and SHPT have been previously linked with fibrosis and the progression of CKD [19]. Recently, moderate reduction of PTH (from 441 to 327 pg/ml) with cinacalcet (10 mg/kg/day) for 12 weeks in the adenine-model of CKD was reported to attenuate the endothelial-to-mesenchymal transition in rat kidneys [10], one of the mechanisms for myofibroblast accumulation in renal fibrogenesis [3, 41]. Cinacalcet was also found to improve kidney histology and reduce creatinine concentration following a protocol where 0.75% adenine diet was given for 4 weeks and then withdrawn for 8 weeks [10]. The untreated adenine-rats still had 10-fold elevation of plasma creatinine at close of the study [10]. Based on these findings, treatment with cinacalcet may help to improve recovery from the adenine-induced kidney insult. In the present study we applied constant 0.3% adenine diet, and in this model cinacalcet was without statistically significant influences on renal function, interstitial inflammation, fibrosis, tubular dilatation, and tubular atrophy.

The present cinacalcet treatment, alone and in combination with sitaxentan, elevated blood hemoglobin concentration by 28%. As there was no significant effect on renal function, the increase in hemoglobin in cinacalcet-treated rats was most probably mediated via the effective PTH suppression. SHPT is known to cause *osteitis fibrosa cystica*, characterized by decreased blood cell formation in the fibrotic bone marrow [42]. Therefore, the lowering of plasma PTH may well explain the partial correction of anemia in rats receiving cinacalcet.

Urinary calcium excretion was increased in all other groups receiving adenine, but not in rats receiving both sitaxentan and cinacalcet. Previously, CRI induced by adenine diet has been found to reduce intestinal Ca^2+^ absorption, increase bone resorption, increase Ca^2+^ incorporation into soft tissues, and enhance urinary Ca^2+^ excretion [5, 43]. The finding that combined treatment with sitaxentan and cinacalcet normalized urinary calcium excretion cannot solely be explained by the improved renal function and reduced calcium efflux from bone via correction of SHPT, as neither of these treatments alone influenced urinary calcium loss. A potential explanation is an effect on bone turnover that is influenced via CaSR and ETA-mediated pathways [44, 45].

Renin is the central rate-limiting enzyme of the RAS [46], and therefore effects on renin expression can shift the balance of the whole system towards vasoconstriction or vasodilatation. In this study, the adenine diet resulted suppressed kidney renin mRNA levels to 45% of the mRNA content in control rats. This was probably caused by the loss of functional kidney tissue. Interestingly, all of the treatments induced a further 40–45% suppression of renin mRNA content, indicating a down-regulation of vasoconstrictive kidney RAS activity. The molecular mechanism of reduced renin mRNA remains unknown, but one putative explanation could be inhibition of human antigen R transcription, an enhancer of renin mRNA stability via interleukin-10 release [47].

Kidney ACE was not significantly affected by the adenine diet, contrary to the surgical remnant kidney model of advanced CKD, in which we have previously found renal ACE to be increased [20, 22]. We have also found that in rat kidney tissue, ACE mRNA content correlates very well with the amount of ACE protein (r~0.8) [20]. In addition, oral calcium supplementation has been found to down-regulate renal ACE mRNA and protein content, but the exact mechanism beyond this reduction has not been discovered [20, 22]. On the basis of the present results with cinacalcet, neither allosteric modulation of the CaSR, nor suppression of PTH, has an influence on ACE mRNA content in renal tissue. Sitaxentan treatment alone slightly reduced renal ACE mRNA content when compared with the Control group, but not when compared with untreated adenine-rats (Ade group). Combined sitaxentan + cinacalcet treatment was without influence on kidney ACE mRNA content, and renal ACE mRNA content did not differ between the groups receiving the adenine diet. Thus, sitaxentan did not have a significant influence on renal ACE mRNA content.

The adenine diet had diverse effects on mRNA levels of RAS components in the kidney, but the most dramatic finding was 94% suppression of renal ACE2 mRNA content when compared with the Control group. ACE2 is considered a protective RAS component, and it degrades angiotensin II and generates angiotensin 1–7 that has vasodilatory, natriuretic and antifibrotic properties [46]. Reduced ACE2 in kidney tissue would shift the balance towards vasoconstrictive and profibrotic RAS activity [46]. Previously, moderate 40% reduction in kidney tissue ACE2 content was reported in the 5/6 nephrectomy rat model of CKD [20]. The present marked suppression of ACE2 following adenine administration raises the possibility that this mechanism is involved in the pathogenesis of kidney damage in this model. Of note, combined ETA blockade with sitaxentan and PTH reduction with cinacalcet moderately but significantly alleviated the suppression of ACE2 in kidney tissue, but neither of these compounds had any significant influences on the mRNA levels of RAS receptors in this study. Thus, parallel ETA receptor stimulation and high PTH level seem to contribute to the harmful RAS activation particularly via suppression of ACE2 in this model.

The adenine diet did not influence kidney AT_4R_ mRNA contents, but reduced those of AT_1aR_, AT_2R_, Mas, and PRR by 40–60%. AT_1aR_ mediates the potentially harmful effects of angiotensin II, so reduced content of this receptor in the kidney could be considered beneficial. The effects of AT_2R_ stimulation seem to mediate vasodilatory and antiproliferative effects [46]. Thus, down-regulation of AT_2R_ might participate in the renal pathophysiology of the adenine model. Down-regulation of the renal AT_2R_ has been previously reported in the 5/6 nephrectomized rat model of CRI [20]. Should the reductions in Mas and PRR mRNA reflect changes at the protein level, these changes may also have contributed to the pathology in the adenine model, as Mas receptor mediates the effects of the antifibrotic angiotensin 1–7 [46, 48], while PRR is thought to be essential for normal podocyte function [49].

The fact that renal fibrosis was not reduced by the present treatments corresponds to the finding that neither sitaxentan nor cinacalcet showed major influences on RAS components or TGF-ß1 levels, two interconnected promoters of interstitial fibrosis [50]. Although there was some variation between the PCR and western blotting results of TGF-ß1, both methods showed increased TGF-ß1 in the adenine model that was not decreased by sitaxentan or cinacalcet. TGF-ß1 induces fibrosis by stimulating epithelial-to-mesenchymal transition, proliferation of fibroblasts, synthesis of extracellular matrix components, and by reducing collagenase production [51]. It has also indirect effects through other profibrotic factors, of which CTGF influences both cellular proliferation and extracellular matrix accumulation [23, 51, 52]. In addition, CTGF enhances TGF-ß1 signaling by 1) binding to the growth factor and supporting its interaction with the TGF-ß1 receptor, 2) affecting other down-stream proteins in TGF-ß1 signaling, and 3) inhibiting the counteracting bone morphogenetic protein 7 (BMP-7) pathway [23, 53]. CTGF expression is stimulated by several other factors besides TGF-ß1, and it can also exert TGF-ß1 independent actions [54].

CTGF mRNA was increased in the adenine model, whereas both sitaxentan and cinacalcet treatments prevented this change. The plausible underlying mechanisms are multiple, including direct antifibrotic effects of the therapies, decreased cholesterol levels, and control of SHPT. The findings on CTGF may be of importance, as it is one of the potential treatment targets in kidney fibrosis [23, 54]. Reduced CTGF is a plausible mechanism for the beneficial morphological findings, as sitaxentan and cinacalcet in combination improved interstitial inflammation, tubular dilatation and tubular atrophy in this model of CRI. However, hemizygous deletion of CTGF did not prevent fibrosis in an advanced nephropathy model in mice. Thus, the significance of CTGF may be overruled by other factors in the fibrotic response during chronic kidney damage, when even normal amounts of CTGF may promote fibrosis [55]. Interestingly, CTGF is a possible independent predictor of end-stage renal disease and mortality in patients with type 1 diabetic nephropathy [54].

## Conclusions

In the adenine rat model of chronic interstitial nephritis, sitaxentan treatment moderately improved kidney function and alleviated tubular atrophy, while combined sitaxentan + cinacalcet improved both kidney function and several indices of tubulointerstitial damage. In the present model, the mRNA and protein contents of kidney tissue TGF-ß1 increased, CTGF mRNA increased, those of ACE and AT_4R_ remained unchanged, while mRNAs of renin, AT_1aR_, AT_2R_, Mas, and PRR were reduced by 40–60%, and that of ACE2 was suppressed by 94%. ETA antagonism with sitaxentan reduced plasma phosphate and PTH concentrations, and reduced kidney CTGF mRNA content. Sitaxentan and cinacalcet, alone and in combination, further reduced kidney renin mRNA by 40% when compared with the Ade group. Cinacalcet was without effect on renal function but significantly reduced plasma PTH, decreased kidney CTGF mRNA, and increased blood hemoglobin. Combined treatment with sitaxentan and cinacalcet increased renal tissue ACE2 mRNA content and normalized urinary calcium losses in the adenine rat model of CRI.

## Additional file

Additional file 1: Figure S1. Representative original blot of TGF-ß1 Western blotting. (PDF 163 kb)

## Abbreviations

ACE: Angiotensin converting enzyme
Ade: 0.3% adenine diet
ANOVA: Analysis of variance
AT_1aR_: Angiotensin II receptor type 1_a_
AT_2R_: Angiotensin II receptor type 2
AT_4R_: Angiotensin IV receptor
BP: Blood pressure
CaSR: Calcium sensing receptor
CKD: Chronic kidney disease
CRI: Chronic renal insufficiency
CTGF: Connective tissue growth factor
ESRD: End stage renal disease
ETA: Endothelin receptor A
PRR: (pro)renin receptor
PTH: Parathyroid hormone
RAS: Renin-angiotensin system
SHPT: Secondary hyperparathyroidism
