# Supplementary material for: Endothelin A receptor blocker and calcimimetic in the adenine rat model of chronic renal insufficiency
Source: BMC Nephrol. 2017 Oct 27;18:323. doi: 10.1186/s12882-017-0742-z (PMC5659028; doi:10.1186/s12882-017-0742-z)
Supplement: Additional file 1: Figure S1. — Representative original blot of TGF-ß1 Western blotting. (PDF 163 kb) [file 12882_2017_742_MOESM1_ESM.pdf]

Endothelin A Receptor Blocker and Calcimimetic in the Adenine Rat Model of Chronic Renal Insufficiency

Suvi Törmänen[a], Ilkka Pörsti[a,b] Päivi Lakkisto[c,d], Ilkka Tikkanen[c,e], Onni Niemelä[a,f], Timo Paavonen[a,g], Jukka Mustonen[a,b], and Arttu Eräranta[a]

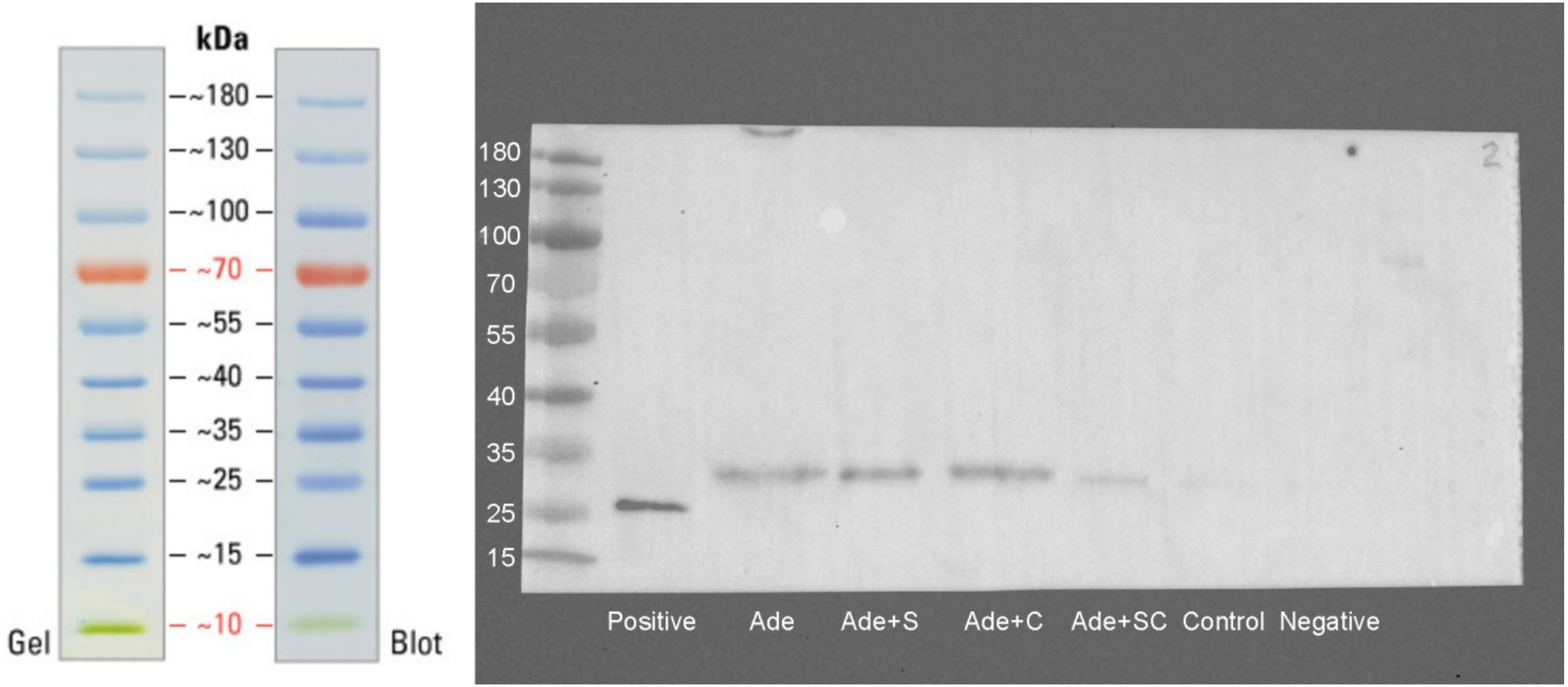

Additional Figure 1. Representative original blot of TGF-ß1 Western blotting; study groups as in Figure 1. Thermo Page Ruler 26616 was used as molecular weight marker, recombinant human TGF-ß1 (R&D cat nro 240-B) was used as positive control in non-reducing conditions, and rat heart tissue was used as negative control. The observed ~2kDa difference in molecular weight is due to interspecies variation in TGF-ß1 protein.
